# Ethanolamine Is a New Anti-Prion Compound

**DOI:** 10.3390/ijms222111742

**Published:** 2021-10-29

**Authors:** Keiji Uchiyama, Hideyuki Hara, Junji Chida, Agriani Dini Pasiana, Morikazu Imamura, Tsuyoshi Mori, Hanae Takatsuki, Ryuichiro Atarashi, Suehiro Sakaguchi

**Affiliations:** 1 Division of Molecular Neurobiology, The Institute for Enzyme Research (KOSOKEN), Tokushima University, 3-18-15 Kuramoto, Tokushima 770-8503, Japan; ku200@tokushima-u.ac.jp (K.U.); hara@tokushima-u.ac.jp (H.H.); jchida@tokushima-u.ac.jp (J.C.); agrianipasiana@yahoo.com (A.D.P.); 2 Division of Genome Medicine, Institute of Advanced Medical Sciences, Tokushima University, Tokushima 770-8503, Japan; 3 Division of Microbiology, Department of Infectious Diseases, Faculty of Medicine, University of Miyazaki, 5200 Kihara, Kiyotake, Miyazaki 889-1692, Japan; morikazu_imamura@med.miyazaki-u.ac.jp (M.I.); tsuyoshi_mori@med.miyazaki-u.ac.jp (T.M.); hanae_takatsuki@med.miyazaki-u.ac.jp (H.T.); ryuichiro_atarashi@med.miyazaki-u.ac.jp (R.A.)

**Keywords:** prions, prion protein, protein misfolding, neurodegeneration, ethanolamine, therapy

## Abstract

Prion diseases are a group of fatal neurodegenerative disorders caused by accumulation of proteinaceous infectious particles, or prions, which mainly consist of the abnormally folded, amyloidogenic prion protein, designated PrP^Sc^. PrP^Sc^ is produced through conformational conversion of the cellular isoform of prion protein, PrP^C^, in the brain. To date, no effective therapies for prion diseases have been developed. In this study, we incidentally noticed that mouse neuroblastoma N2a cells persistently infected with 22L scrapie prions, termed N2aC24L1-3 cells, reduced PrP^Sc^ levels when cultured in advanced Dulbecco’s modified eagle medium (DMEM) but not in classic DMEM. PrP^C^ levels remained unchanged in prion-uninfected parent N2aC24 cells cultured in advanced DMEM. These results suggest that advanced DMEM may contain an anti-prion compound(s). We then successfully identified ethanolamine in advanced DMEM has an anti-prion activity. Ethanolamine reduced PrP^Sc^ levels in N2aC24L1-3 cells, but not PrP^C^ levels in N2aC24 cells. Also, oral administration of ethanolamine through drinking water delayed prion disease in mice intracerebrally inoculated with RML scrapie prions. These results suggest that ethanolamine could be a new anti-prion compound.

## 1. Introduction

Prion diseases, or transmissible spongiform encephalopathies, are a group of fatal neurodegenerative disorders in humans and animals, caused by accumulation of proteinaceous infectious particles, the so-called prions, in the brain [1,2]. Prions are widely believed to consist of the protease-resistant, amyloidogenic isoform of prion protein, designated PrP^Sc^, which is generated through conformational conversion of the normal cellular isoform of prion protein, PrP^C^. PrP^C^ is a host-encoded membrane glycoprotein tethered to the plasma membrane via a glycosylphosphatidylinositol (GPI) anchor moiety and expressed most abundantly in the brain, particularly by neurons, and to a lesser extent in non-neuronal tissues [1,2]. We and others have shown that mice devoid of PrP^C^ (*Prnp^0/0^*) were resistant to prion infection, neither propagating PrP^Sc^ or prions in their brains nor developing disease even after intracerebral inoculation with prions [3,4,5,6], reinforcing that the conversion of PrP^C^ into PrP^Sc^ leading to the accumulation of PrP^Sc^ in the brain is a key pathogenic event in prion diseases.

Prion diseases in humans manifest as sporadic, genetic, and acquired forms, with an annual incidence of 1:1,000,000 worldwide [2]. Sporadic Creutzfeldt–Jakob disease (sCJD) is the most common human prion disease, accounting for 85–90% of cases [2]. The etiologies of sCJD are unknown. Genetic prion diseases, which include familial CJD, Gerstmann–Sträussler–Scheinker syndrome, and fatal familial insomnia, are causatively linked to specific mutations in the prion protein gene and constitute 10–15% of cases [2]. The remaining cases are those of acquired prion diseases, caused by intra- or inter-species infection with prions [2]. These include iatrogenic CJD transmitted through medical treatments or procedures [7,8,9,10], kuru in Papua New Guinea spread by ritualistic cannibalism [11], and variant CJD transmitted from bovine spongiform encephalopathy (BSE) via contaminated food [12,13,14]. No effective therapies against prion diseases have yet been developed. Identification of anti-prion compounds, which can reduce PrP^Sc^ in prion-infected neurons, is thus useful for the development of therapies against prion diseases.

In the present study, we incidentally noticed that advanced Dulbecco’s modified eagle medium (DMEM), which has been developed to allow cell cultures with reduced supplementation of fetal bovine serum (FBS), contains an anti-prion compound(s) that is capable of reducing PrP^Sc^ levels in prion-infected cells, and we subsequently identified that ethanolamine in advanced DMEM has an anti-prion activity. We also show that oral administration of ethanolamine delayed prion disease in prion-infected mice. These results suggest that ethanolamine could be a new anti-prion compound.

## 2. Results

### 2.1. Advanced DMEM Contains an Anti-Prion Compound(s)

**Figure 1:**
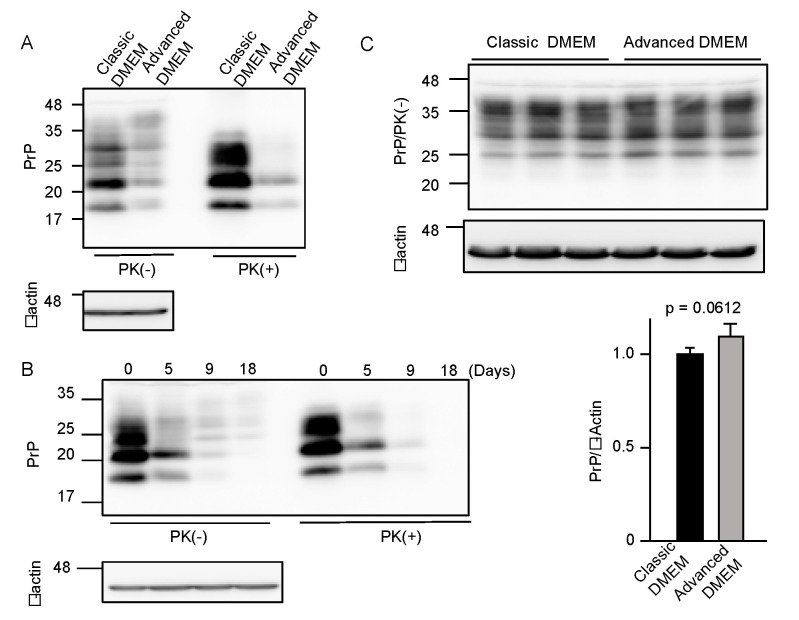
Advanced DMEM reduces PrP^Sc^ levels in prion-infected cells. (**A**) Western blotting with 6D11 anti-PrP antibody of proteinase K (PK)-treated or -untreated cell lysates from prion-infected N2aC24L1-3 cells cultured in classic or advanced DMEM for 6 days. β-actin is an internal control. (**B**) Western blotting with 6D11 anti-PrP antibody of PK-treated or -untreated cell lysates from N2aC24L1-3 cells cultured in advanced DMEM for the indicated days. β-actin is an internal control. (**C**) Upper panels: Western blotting with 6D11 anti-PrP antibody of triplicate cell lysates from prion-uninfected N2aC24 cells cultured in classic or advanced DMEM for 16 days. β-actin is an internal control. Lower panels: Densitometric analysis of PrP^C^ levels in the left panels.

To investigate if advanced DMEM is as available for prion-infected cell cultures as classic DMEM, we cultured 22L scrapie prion-infected mouse neuroblastoma N2a cells, termed N2aC24L1-3 cells [15], in advanced DMEM with 2% FBS and in classic DMEM with 10% FBS, and compared PrP^Sc^ levels in the cell lysates by Western blotting with 6D11 anti-PrP antibody, which recognizes residues 97–100 of mouse PrP [16]. The proteinase K (PK)-resistant fragments of PrP^Sc^ were reduced in N2aC24L1-3 cells cultured in advanced DMEM with 2% FBS, compared to those in classic DMEM with 10% FBS (Figure 1A). PrP^Sc^ levels in N2aC24L1-3 cells were further reduced to an almost undetectable level at 18 days post-culture in advanced DMEM with 2% FBS (Figure 1B). In contrast, PrP^C^ levels were unchanged in prion-uninfected parent N2aC24 cells [15] even at 16 days post-culture in advanced DMEM with 2% FBS (Figure 1C). These results suggest that advanced DMEM may contain an anti-prion compound(s) that is able to reduce PrP^Sc^ levels without affecting PrP^C^ levels in prion-infected cells.

### 2.2. Ethanolamine Is an Anti-Prion Compound in Advanced DMEM

**Figure 2:**
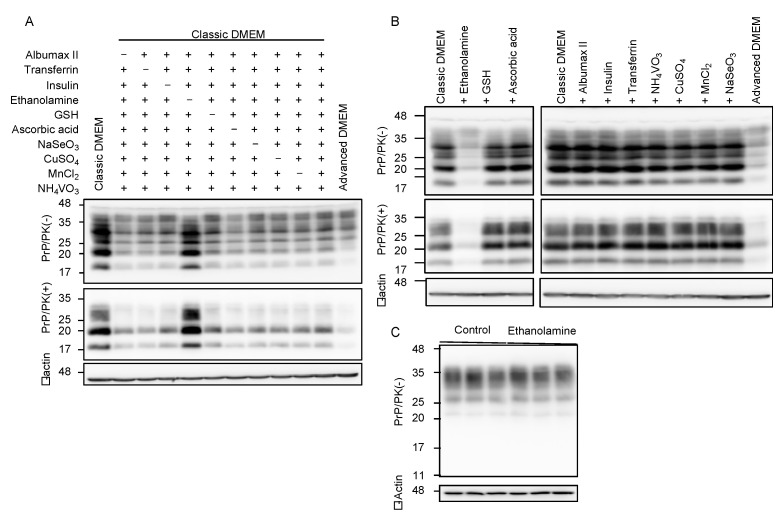
Ethanolamine reduces PrP^Sc^ levels in prion-infected cells. (**A**) Western blotting with 6D11 anti-PrP antibody of PK-treated or -untreated cell lysates from N2aC24L1-3 cells cultured in classic DMEM supplemented with various combinations of indicated compounds for 8 days. β-actin is an internal control. Ethanolamine, 1.9 mg/L (31 μM); GSH, 1 mg/L; ascorbic acid phosphate, 2.5 mg/L; recombinant insulin, 10 mg/L; human transferrin (holo), 7.5 mg/L; AlbuMAX II, 400 mg/L; Na_2_SeO_3_, 0.005 mg/L; NH_4_VO_3_, 0.0003 mg/L; CuSO_4_, 0.00125 mg/L; MnCl_2_, 0.00005 mg/L. (**B**) Western blotting with 6D11 anti-PrP antibody of PK-treated or -untreated cell lysates from N2aC24L1-3 cells cultured in classic DMEM supplemented with various combinations of indicated compounds for 10 days. β-actin is an internal control. Ethanolamine, 1.9 mg/L; GSH, 1 mg/L; ascorbic acid phosphate, 2.5 mg/L; recombinant insulin, 10 mg/L; human transferrin (holo), 7.5 mg/L; AlbuMAX II, 400 mg/L; Na_2_SeO_3_, 0.005 mg/L; NH_4_VO_3_, 0.0003 mg/L; CuSO_4_, 0.00125 mg/L; MnCl_2_, 0.00005 mg/L. (**C**) Western blotting with 6D11 anti-PrP antibody of cell lysates from N2aC24 cells cultured in classic DMEM supplemented with or without ethanolamine (1.8 mg/L, 30 μM) for 6 days. β-actin is an internal control.

Compared to classic DMEM, advanced DMEM contains 10 more compounds, which are ethanolamine, glutathione (GSH), ascorbic acid, insulin, transferrin, lipid-rich bovine serum albumin (BSA, AlbuMax II), and the trace elements of sodium selenite (Na_2_SeO_3_), ammonium metavanadate (NH_4_VO_3_), cupric sulfate (CuSO_4_), and manganous chloride (MnCl_2_). To identify the anti-prion compound(s) that may be included in advanced DMEM, we cultured N2aC24L1-3 cells in classic DMEM with 10% FBS supplemented with various combinations of the nine compounds out of 10 compounds, and investigated the cell lysates for PrP^Sc^ levels by Western blotting with 6D11 anti-PrP antibody. All the classic DMEMs with ethanolamine supplementation reduced PrP^Sc^ levels in N2aC24L1-3 cells (Figure 2A). In contrast, only the classic DMEM without ethanolamine supplementation failed to reduce PrP^Sc^ levels in N2aC24L1-3 cells (Figure 2A). These results suggest that ethanolamine could be the anti-prion compound included in advanced DMEM. To further confirm the anti-prion activity of ethanolamine, we cultured N2aC24L1-3 cells in classic DMEM with 10% FBS supplemented with each of the 10 compounds, including ethanolamine. Western blotting with 6D11 anti-PrP antibody showed that supplementation with ethanolamine, but not with the other nine compounds, in classic DMEM reduced PrP^Sc^ levels in N2aC24L1-3 cells (Figure 2B). PrP^C^ levels were not reduced in N2aC24 cells cultured in classic DMEM with 10% FBS supplemented with ethanolamine (Figure 2C). These results indicate that ethanolamine is the anti-prion compound included in advanced DMEM.

### 2.3. Ethanolamine Delays Prion Disease in Prion-Infected Mice

**Figure 3:**
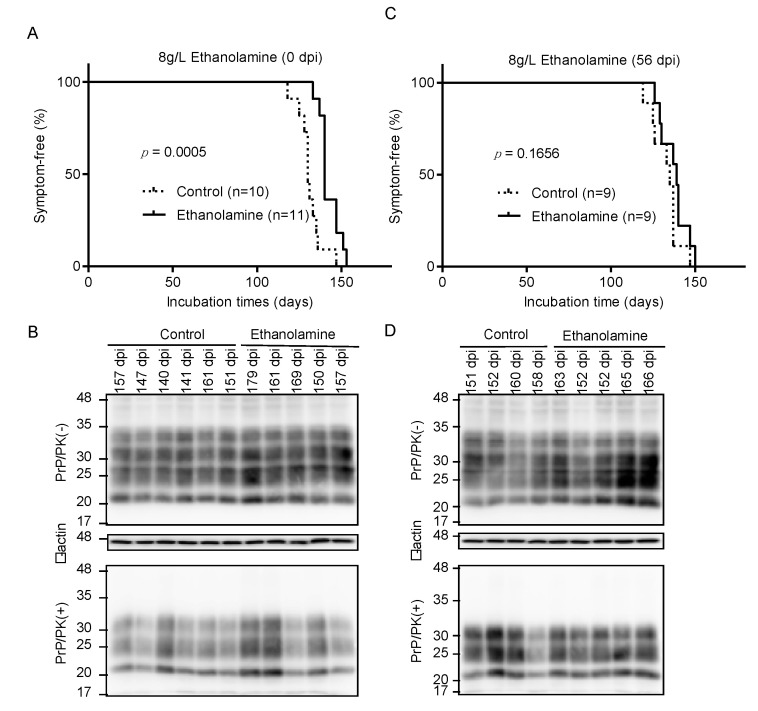
Ethanolamine delays prion disease in prion-infected mice. (**A**) Percentage of symptom-free mice intracerebrally inoculated with RML prions after oral administration with or without ethanolamine through drinking water starting from the inoculation day. (**B**) Western blotting with 6D11 anti-PrP antibody of PK-treated or -untreated brain homogenates from the control (*n* = 6) and ethanolamine-administrated, ill mice (*n* = 5) in (**A**). The mice were sacrificed at the indicated dpi. β-actin is an internal control. (**C**) Percentage of symptom-free mice intracerebrally inoculated with RML prions after oral administration with or without ethanolamine through drinking water starting from 56 dpi. (**D**) Western blotting with 6D11 anti-PrP antibody of PK-treated or -untreated brain homogenates from the control (*n* = 4) and ethanolamine-administrated, ill mice (*n* = 5) in (**C**). The mice were sacrificed at the indicated dpi. β-actin is an internal control.

To investigate if ethanolamine could exert its anti-prion activity in vivo, we intracerebrally inoculated RML scrapie prions into mice and orally administrated them with ethanolamine (8 g/L) through drinking water ad libitum from 0 days post-inoculation (dpi). Control mice were similarly given ethanolamine-free water. Control mice developed disease at 130 ± 5 dpi, with disease-specific symptoms, such as weight loss, decreased locomotive activity, ruffled hair coat, and hunched back (Figure 3A). However, ethanolamine-administrated mice exhibited significantly longer incubation times of 143 ± 6 dpi (*p* = 0.0005) (Figure 3A). Western blotting with 6D11 anti-PrP antibody of control and ethanolamine-administrated, ill mice showed similar accumulation of PrP^Sc^ in their brains (Figure 3B), suggesting that ethanolamine does not affect the final accumulation levels of PrP^Sc^ in the brain of prion-infected mice. To further investigate the anti-prion activity of ethanolamine in vivo, we intracerebrally inoculated mice with RML prions and orally administrated them with ethanolamine (8 g/L) from 56 dpi. Control mice were also given ethanolamine-free water. The incubation times of ethanolamine-administrated mice were extended, but not significantly, compared to those of control mice (138 ± 8 vs. 133 ± 8 dpi, *p* = 0.1656) (Figure 3C). Western blotting showed similar levels of PrP^Sc^ in the brains of control and ethanolamine-administered, ill mice (Figure 3D). These results indicate that ethanolamine is effective against prion infection in vivo, delaying prion disease in prion-infected mice in a manner dependent on the timing of its administration.

### 2.4. The Anti-Prion Activity of Ethanolamine Is Dose-Dependent

**Figure 4:**
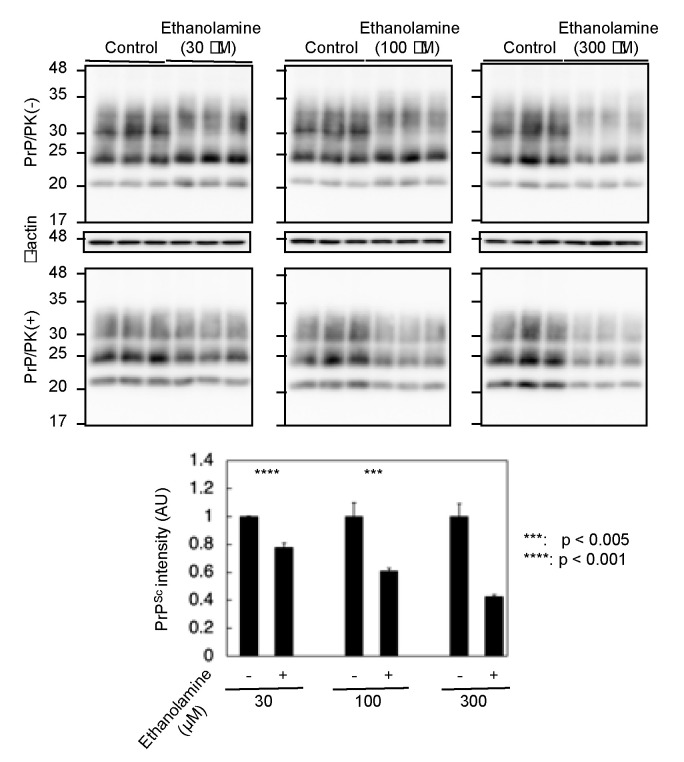
The anti-prion activity of ethanolamine is dose-dependent. Western blotting with 6D11 anti-PrP antibody of cell lysates from N2aC24L1-3 cells cultured in classic DMEM supplemented with 30 μM (1.83 mg/L), 100 μM (6.11 mg/L), and 300 μM (18.3 mg/L) of ethanolamine for 6 days. The densities of the PK-resistant fragments of PrP^Sc^ were statistically analyzed.

To gain insight into the mechanism of the anti-prion activity of ethanolamine, we investigated if ethanolamine could have increased anti-prion activity in a dose-dependent way. N2aC24L1-3 cells were cultured in classic DMEM with 10% FBS together with different doses of ethanolamine and subjected the cell lysates into Western blotting with 6D11 anti-PrP antibody. The PK-resistant PrP fragments of PrP^Sc^ were reduced in N2aC24L1-13 cells after treatment with ethanolamine in a dose-dependent manner (Figure 4). These results indicate that the anti-prion activity of ethanolamine could be dose-dependent.

### 2.5. Ethanolamine Does Not Affect the Localization of PrP^C^ at Lipid Rafts

**Figure 5:**
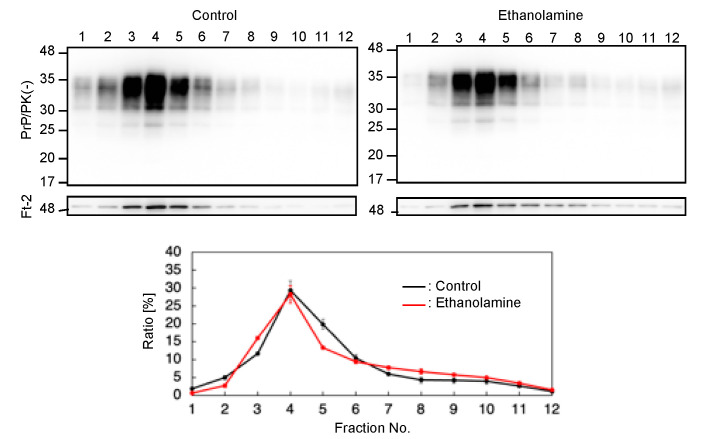
Ethanolamine does not affect the localization of PrP^C^ at lipid rafts. Upper panels: Western blotting with 6D11 anti-PrP antibody of cell lysates from N2aC24 cells cultured in classic DMEM supplemented with ethanolamine (100 μM) for 6 days after fractionation by the sucrose density gradient assay. Flotillin-2 is the lipid raft marker. Lower panel: Densitometric analysis of PrP^C^ levels from triplicate experiments.

PrP^C^ predominantly localizes on the plasma membrane, particularly at lipid raft domains, which has been suggested to be one of the major subcellular sites for the conversion of PrP^C^ into PrP^Sc^ [17,18]. We thus investigated if ethanolamine could affect the subcellular localization of PrP^C^ at lipid raft domains. We cultured N2aC24 cells in classic DMEM with 10% FBS together with ethanolamine and subjected the cell lysates to a sucrose density gradient assay to assess the localization of PrP^C^ at lipid raft domains. PrP^C^ was predominantly detected at the raft domain fractions, which were represented by the presence of the raft protein, flotillin-2, in both ethanolamine-treated and -untreated N2aC24 cells (Figure 5). These results indicate that ethanolamine does not affect the subcellular localization of PrP^C^ at raft domains.

### 2.6. Ethanolamine Does Not Disturb In Vitro Conversion of PrP^C^ into PrP^Sc^

**Figure 6:**
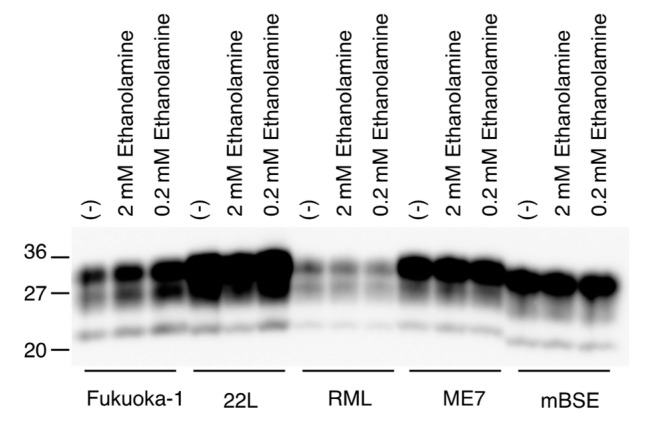
Ethanolamine does not disturb in vitro conversion of PrP^C^ into PrP^Sc^. Western blotting of PMCA products performed with or without ethanolamine after treatment with PK.

We also investigated if ethanolamine could directly affect the conversion of PrP^C^ into PrP^Sc^, by using a protein misfolding cyclic amplification (PMCA) technique, which has been developed to amplify PrP^Sc^ by inducing the conversion of PrP^C^ into PrP^Sc^ in vitro [19]. Mouse brain homogenates infected with various mouse-adapted prions, including Fukuoka-1, 22L, RML, ME7, and mBSE prions, were mixed with normal mouse brain homogenates and subjected to a first round of PMCA with or without ethanolamine. The resulting PMCA products were treated with PK and investigated for PrP^Sc^ production by Western blotting with anti-PrP HRP-conjugated monoclonal antibody T2 [20], which recognizes residues 135-140 of mouse PrP. All prion strains used converted PrP^C^ into PrP^Sc^ in PMCA in the absence of ethanolamine, but to different degrees in a strain-dependent way (Figure 6). Even a high dose of ethanolamine did not impair the activity of each prion strain to convert PrP^C^ into PrP^Sc^ in PMCA (Figure 6). These results suggest that ethanolamine might not directly disturb the conversion of PrP^C^ into PrP^Sc^.

## 3. Discussion

In this study, we first showed that PrP^Sc^ levels were reduced in prion-infected N2aC24L1-3 cells when cultured in advanced DMEM. This suggests that advanced DMEM may contain an anti-prion compound(s). We subsequently identified that ethanolamine in advanced DMEM has an anti-prion activity, reducing PrP^Sc^ levels in N2aC24L1-three cells in a dose-dependent manner. PrP^C^ levels were not reduced by ethanolamine in prion-uninfected parent N2aC24 cells. We also showed that ethanolamine was effective against prion infection in vivo, delaying prion disease in RML prion-infected mice when orally administered. These results suggest that ethanolamine is a new anti-prion compound that does not affect PrP^C^ levels in prion-infected cells.

PrP^C^ predominantly locates at the lipid raft domains of the plasma membrane as a GPI-anchored glycoprotein [17]. It has thus been suggested that raft domains could be one of the major sites for the conversion [18]. We showed that ethanolamine did not affect the subcellular localization of PrP^C^ at lipid raft domains in prion-uninfected N2aC24 cells. We also showed that ethanolamine did not disturb the in vitro conversion of PrP^C^ into PrP^Sc^. These results suggest that ethanolamine could reduce PrP^Sc^ levels in prion-infected cells by neither affecting the subcellular localization of PrP^C^ at lipid raft domains nor directly impairing the conversion of PrP^C^ into PrP^Sc^. Ethanolamine is utilized for the synthesis of phosphatidylethanolamine, a major lipid component of cellular membranes [21]. It was reported that phosphatidylethanolamine facilitated the conversion of recombinant PrP into PK-resistant PrP in PMCA [22]. However, other investigators reported that phosphatidylethanolamine functioned as an inhibitor for the conversion in another in vitro conversion assay, termed a real-time quaking-induced conversion assay [23]. Thus, it might be interesting to investigate if phosphatidylethanolamine is an effector molecule for ethanolamine to exert its anti-prion activity in prion-infected cells.

Advanced DMEM has been developed to enable cell cultures with reduced FBS supplementation by adding compounds, including ethanolamine in classic DMEM. We showed that ethanolamine in advanced DMEM is an anti-prion compound, reducing PrP^Sc^ levels in prion-infected cells and delaying prion disease in prion-infected mice. Therefore, advanced DMEM is not available for the cultures of prion-infected cells. However, it remains to be investigated whether advanced DMED depleted of ethanolamine could retain the activity to support cell cultures with reduced FBS supplementation for not only prion-infected cells but also other cells.

In short, we found that ethanolamine is a new anti-prion compound able to reduce PrP^Sc^ levels in prion-infected cells and delay prion disease in prion-infected mice. Elucidation of the mechanism of the anti-prion activity of ethanolamine could be valuable not only for understanding of the conversion mechanism of PrP^C^ into PrP^Sc^ but also for the development of therapeutics against prion diseases.

## 4. Materials and Methods

### 4.1. Ethics Statement

The Ethics Committee of Animal Care and Experimentation of Tokushima University approved the animal experiments in this study (approval number T2021-2, 13 April 2021). Animals were cared for in accordance with The Guiding Principle for Animal Care and Experimentation of Tokushima University and guidelines under the jurisdiction of the Ministry of Education, Culture, Sports, Science and Technology, Japan.

### 4.2. Reagents and Antibodies

The following reagents and antibodies used in this study were commercially purchased: classic DMEM (043-30085, Wako Pure Chemical Industries, Osaka, Japan), advanced DMEM (12491-015, Gibco Life Technologies Corporation, Gland Island, NY, USA), proteinase K (165-21043, Wako Pure Chemical Industries), 6D11 mouse anti-PrP Ab (808003, BioLegend, San Diego, CA, USA), mouse anti-ß-actin Ab (M177-3, Medical and Biological Laboratories, Tokyo, Japan), rabbit anti-flottilin-2 Ab (3436, cell signaling technology, Danvers, MA, USA), anti-mouse IgG horseradish peroxidase (HRP)-linked Ab (NA931, GE Healthcare, Little Chalfont, UK), anti-rabbit IgG horseradish peroxidase (HRP)-linked Ab (NA934, GE Healthcare), AlbuMAXII lipid-rich bovine serum albumin (11021029, Thermo Fischer Scientific Inc., Waltham, MA, USA), transferrin (208-18971, Wako Pure Chemical Industries), insulin (15500, Sigma–Aldrich, Co. LLC, St. Louis, MO, USA), ethanolamine (E6133, Sigma–Aldrich, Co. LLC), glutathione (reduced form) (17050-72, Nacalai Tesque, Kyoto, Japan), ascorbic acid (A8960, Sigma–Aldrich, Co. LLC), sodium selenite (11707-04, Nacalai Tesque), cupric sulfate (09605-04, Nacalai Tesque), manganous chloride (139-00722, Wako Pure Chemical Industries), ammonium metavanadate (02705-62, Nacalai Tesque), and FBS (10437-028, Gibco Life Technologies Corporation). HRP-conjugated T2 anti-PrP antibody [20] was kindly provided by Dr. Y. Iwamaru (National Institute of Animal Health, Japan).

### 4.3. Cells and Animals

N2aC24 and N2aC24L1-3 cells [15] were cultured at 37 °C with 5% CO_2_ in air in classic DMEM (Wako Pure Chemical Industries) with 10% FBS (Gibco Life Technologies Corporation) or in advanced DMEM (Gibco Life Technologies Corporation) with 2% FBS (Gibco Life Technologies Corporation) supplemented with 1× Penicillin-Streptomycin Mixed Solution (26253-84, Nacalai Tesque). Crl:CD1(ICR) mice were purchased from Charles River Laboratories Japan (Kanagawa, Japan).

### 4.4. Reagent Treatments in Cell Cultures

Reagents were added in the culture media at indicated concentrations for the indicated days.

### 4.5. Western Blotting

Cells and brain tissues were lysed in a lysis buffer containing 150 mM NaCl, 50 mM Tris-HCl (pH 7.5), 0.5% Triton X-100, 0.5% sodium deoxycholate, and 1 mM EDTA using a Multi-beads shocker (Yasui Kikai Co., Osaka, Japan), and the protein concentrations were determined using the BCA protein assay kit (23225, Pierce, Rockford, IL, USA). After treatment with or without PK (Wako Pure Chemical Industries) at 20 µg/mL for 30 min at 37 °C, total proteins were electrophoresed through an SDS-polyacrylamide gel, and electrically transferred to an Immobilon-P PVDF membrane (IPVH0010, Millipore Corp., Billerica, MA, USA). The membrane was treated in 5% non-fat dry milk-containing TBST (0.1% Tween-20, 100 mM NaCl, 10 mM Tris-HCl, pH7.6) for 1 h at room temperature (RT), and incubated with the first antibodies overnight at 4 °C in 1% non-fat dry milk-containing TBST. The membrane was washed in TBST several times. Signals were visualized using horseradish peroxidase (HRP)-conjugated second antibodies and Immobilon Western chemiluminescent HRP substrate (Millipore). The signals were detected using a chemiluminescence image analyzer, LAS-4000 mini (Fujifilm Co., Tokyo, Japan). Signal densities were measured using Image Gauge software (Fuji Film).

### 4.6. Ethanolamine Treatment in Prion-Infected Mice

One percent brain homogenate in phosphate-buffered saline (PBS) from RML prion-infected, ill ICR mice was intracerebrally inoculated into a 4–5 week-old ICR mouse with its 20 μL aliquot. The mice were orally given water ad libitum with or without ethanolamine (8 g/L) from 0 or 56 dpi. The signs for disease-related symptoms were evaluated as previously described [24].

### 4.7. PMCA Assay

Prion-infected and -uninfected brains from ICR mice were homogenized at 10% in a PMCA buffer including 4 mM EDTA and 1% Triton-X in PBS using a Beads Crusher (TAITEC, Aichi, Japan) and then rotated at 4 °C for 1.5 h. PMCA samples were prepared by adding 5 µL of serially diluted ethanolamine at the indicated final concentrations to 45 μL of the mixtures containing prion-uninfected brain homogenates (10% *w/v*), prion-infected brain homogenates (0.01% *w/v*), 0.3 mg/mL heparin, 0.05% digitonin, and 2 mm zirconia beads (TOMY, Tokyo, Japan) in 0.2 mL 8-strips PCR tubes (Seiko, Fukuoka, Japan). The samples were then positioned on a floated plate holder of a microsonicator (Misonix-3000, Cole-Parmer, Vernon Hills, IL, USA). PMCA was performed for 16 h by repeating a procedure of 29 min 40 sec-incubation at 40 °C and a 20 sec-pulse of sonication. The PMCA products were digested with 40 μg/mL of proteinase K in SDS sample buffer, heated to 95 °C for 10 min and then subjected to Western blotting with anti-PrP HRP-conjugated monoclonal antibody T2 [20].

### 4.8. Sucrose Density Gradient Assay

Cells were rinsed 3 times with PBS, lysed in MBS buffer (25 mM MES-NaOH, pH 6.5, 150 mM NaCl) containing 1% Triton X-100, and homogenized by passing through a 21G-needle 15 times. After centrifugation at 500× *g* for 5 min at 4 °C, protein concentration in the lysate was determined using a BCA protein assay kit (Pierce) and adjusted to 10 mg of protein/mL with MBS buffer. 400 µL of the lysates were mixed with 400 µL of MBS buffer containing 80% (*w/v*) sucrose to make 40% (*w/v*) sucrose solution. 600 µL of the solution were then placed at the bottom of the ultra-centrifugation tube (332245A, Hitachi Koki, Tokyo, Japan) containing 1800 µL of 30% (*w/v*) sucrose in MBS buffer and 600 µL of 5% (*w/v*) sucrose in MBS buffer. Samples were centrifuged at 300,000× *g* for 33 h at 4 °C in a P50S2 rotor (Hitachi Koki, Tokyo, Japan). Twelve fractions (150 µL/fraction) and four fractions (300 µL/fraction) were collected from the top and subjected to Western blotting analysis.

### 4.9. Statistical Analysis

Incubation times were analyzed using the log-rank test. Signal densities were analyzed using the Student’s *t*-test.
